# Could more innovation output bring better financial performance? The role of financial constraints

**DOI:** 10.1186/s40854-021-00309-2

**Published:** 2022-01-10

**Authors:** Benlu Hai, Ximing Yin, Jie Xiong, Jin Chen

**Affiliations:** 1 Henan Normal University, Xinxiang, China; 2 Beijing Institute of Technology, 5 South Zhongguancun Street, Haidian District, Beijing, 100081 China; 3 ESSCA School of Management, 1 Rue Joseph Lakanal, Angers, 49000 France; 4 Tsinghua University, Beijing, China

**Keywords:** Innovation output, Financial performance, Individual financial constraints, Market-based financial constraints, PFI framework

## Abstract

Innovation scholars highlight the economic benefits to firms, while research findings on the relationship between innovation output and economic returns remain mixed. In this study, we develop the profiting from innovation (PFI) framework and address the crucial role of financial constraints in the relationship between innovation output and financial performance. We argue that the liability of newness differentiates firms’ financial performance during the commercialization of innovation, leading to a U-shaped relationship between firms’ innovation output and financial performance. We further document the moderating impact of individual financial constraints (IFC) and market-based financial constraints (MFC) on this curvilinear relationship. Empirical tests based on the 142,972 firm-year observations of the multi-source dataset of Chinese manufacturing firms from 1999–2009 support our hypotheses. The additional analysis shows that non-state-owned enterprises and small and medium enterprises benefit more from the synergistic effect of reductions of IFC and MFC than state-owned enterprises and large firms. Our study enriches the literature of the PFI framework by uncovering the mechanism between innovation output and economic returns where financial constraints play an essential role. To the best of our knowledge, we are among the first to investigate the processes and mechanisms between innovation output and financial performance, generating novel insights for business practitioners and policymakers.

## Introduction

Innovation is widely regarded as the engine of growth and long-term economic development (Arrow 1972; Franko 2010; Romer 1986, 1989) for the knowledge economy. However, innovators often fail to obtain potential economic returns due to the uncertain nature of innovation processes and their market outcomes (Hall et al. 2015; Hottenrott and Peters 2012). Scholars recognize such dilemmas and have tried to address such problems theoretically. For instance, the “Profiting from Innovation” (PFI) framework explains why innovating firms often fail to obtain significant economic returns from innovation (Teece 1986). The PFI framework enveloped a far broader array of factors than had hitherto been addressed in the economic analysis of innovation (Pisano 2006; Teece 2018, 2010).

The PFI framework represented a significant break from industrial organization tradition. However, it failed to explore the role of finance as the implicit assumption was that risk capital was available from a company’s balance sheet, the venture capital community, alliance partners, or commercial banks (Teece 2006). There is a long tradition, going back to Schumpeter and beyond, emphasizing the importance of access to risk capital. Financial constraints are now broadly recognized as the significant barrier to innovation and commercialization (Cincera and Ravet 2010). The different dimensions of innovation activities, including innovation input (e.g., R&D investment), intermediary output (e.g., patents), and the final output (new products or services), are all associated with different costs and risks; thus, the access to risk capital, whether from internal or external sources, plays an essential role in the process of innovation (Fagerberg et al. 2005; Mina et al. 2013).

Some established studies have tried to examine how financial constraints affect the relationship between innovation and firm performance. However, the implications of studies such as “more money, more innovation” (Brown et al. 2012), “less money, better innovation” (Almeida et al. 2013; Musso and Schiavo 2008), and “more innovation, less money” (Hottenrott and Peters 2012; Mina et al. 2013) make this issue remain controversial. The mixed results call for further studies on financial constraints in the relationship between innovation activities and firm performance. Moreover, while most research focuses either on the impact of innovation inputs or intermediary output on firm performance, the link between the final innovation output and firm performance remains unclear and underexplored.

In this study, we address this gap from a holistic innovation-financial output logic to open the “black box” between innovation output and firm financial performance by unraveling the role of financial constraints. Specifically, we ask two related sub-questions: could more innovation output bring better economic returns? Do financial constraints matter? To answer these questions, we propose a theoretical framework that explains how financial constraints affect the relationship between innovation output and financial performance. We empirically address this important but understudied topic based on a multi-source dataset of 142,972 firm-year observations of Chinese manufacturing firms from 1999 to 2009.

Our work contributes to the innovation literature in four ways. First, we identify the relationship between innovation output and firms’ financial performance as a U-shaped function due to the liability of newness (Gimenez-Fernandez et al. 2020; Stinchcombe 1965; Yang and Aldrich 2017) and complementary asset investment (Rothaermel 2001) for commercializing innovation outputs, contributing to the PFI framework (Teece 1986) and innovation literature (Arrow 1972; Franko 2010; Romer 1986, 1989) with empirical evidence and curvilinear relationship. Second, we propose a new way to measure financial constraints by differentiating the external financial environment from internal financial sources. This separation has mostly been ignored by the current literature (Cincera and Ravet 2010). Third, we further differentiate the role of individual financial constraints from market-based financial constraints and examine both their standalone and joint moderating effects on the relationship between innovation output and financial performance. This allows us to understand the impact of financial constraints on innovative activities (Yin et al. 2019). Last, China is an ideal context to study the impact of finance on innovation and profiting from innovation as it is transiting from a central-planned economy into a market-based one in which overcoming financial exclusion and constructing a more mature financial market is the key to its economic growth (Gordon and Li 2003). Our research helps to generate novel insights on nourishing innovation-driven development in emerging markets.

## Theoretical developments and hypotheses

### The dimensions of financial constraints

Established literature highlights the importance of financial constraints on firm performance and demands attention to the measurements of such constraints. To measure financial constraints, scholars have proposed indicators of investment-cash-flow sensitivities (Fazzari et al. 1988), the Kaplan and Zingales (KZ) index (Kaplan and Zingales 1997), the Whited and Wu (WW) index (Whited and Wu 2006), SA index of constraints (Hadlock and Pierce 2010), and other sorting criteria based on firm characteristics (Fee et al. 2009). Additionally, existing research has also revealed the factors affecting financial constraints, such as firm size and age (Berger and Udell 2002; Czarnitzki 2006; Czarnitzki and Hottenrott 2011; Himmelberg and Petersen 1994; Petersen and Rajan 1995), governance structures (Chung and Wright 1998; Czarnitzki and Kraft 2004), industry patterns (Bloch 2005; Hall 1992), and financial market regimes (Baum et al. 2009; Bhagat and Welch 1995; Bond et al. 2005; Hall et al. 1998).

Financial data distributions are inherently complex (Li et al. 2021). Despite the different methods to measure financial constraints, most rely on endogenous financial choices, insufficient to measure financial constraints (Hadlock and Pierce 2010). Therefore, our study differentiates the firm’s financial constraints based on their source of origin to separate internal factors from external ones. Specifically, we define individual financial constraints (IFC) as the internal constraints caused by firm-specific characteristics, such as firm size, age, governance structure, and industry pattern. (Bloch 2005; Czarnitzki and Hottenrott 2011; Czarnitzki and Kraft 2004; Hall 1992). Moreover, we define market-based financial constraints (MFC) as the external constraints originating from the underdevelopment and imperfection of the financial market, such as the development level of the banking system, capital market, and equity market (Brown et al. 2012; Chemmanur et al. 2014; Cornaggia, Mao, Tian, & Wolfe, 2012). This separation allows us to take a holistic view of the measurement of financial constraints and understand the mechanisms of different financial constraints in the correlation between innovation output and firm performance.

### Main effect of innovation output on financial performance

Innovative activities may allow innovators to earn monopoly profits (Lieberman and Montgomery 2010; Schumpeter 1979). Numerous studies have validated the positive relationship between innovation and firm performance (Cho and Pucik 2005; Roberts 1999)*.* However, firm-level profits may not follow the same pattern (Artz et al. 2010). The initial high returns from new products gradually decrease due to increasing competition, more entrants, the defensive strategy of incumbents, and market share shrinkage (Pisano and Teece 2007). Firms aiming to maintain the success of new products or services in the market often face the dilemma of choosing between market success (i.e., market share) and financial success (i.e., profits).

Significantly, most new product producers initially suffer from the liability of newness (Gimenez-Fernandez et al. 2020; Stinchcombe 1965; Yang and Aldrich 2017), termed by Stinchcombe (1965), arguing that the emerging organizations and new technologies or products face complex challenges limiting their viability, including lack of legitimacy, managing relationships among strangers, assembling resources quickly (Yang and Aldrich 2017), and pressures from incumbent organizations (Gimenez-Fernandez et al. 2020). The liability of newness often leaves these innovating firms in a position of little competitive advantage and influence over the market, followed by less competitiveness (Kor and Misangyi 2008; Romanelli 1989) and a relatively small proportion of profit from their innovation outputs (Lee et al. 2021), i.e., new products. Due to the lack of history and presence in the market, innovating firms are compelled to signal legitimacy to establish reliable exchange relationships (Hannan and Freeman 1984) and market alliances (Rothaermel 2001). Lacking the safety network and trust of familiar partners, innovating firms are vulnerable to opportunism and endure precariousness in the relationships they seek (Morse et al. 2010; Stinchcombe 2000). Hence, innovating firms that introduce new products into the market have to invest more in marketing activities, especially channel management and even new platforms, to compete with the incumbent players and occupy the market quickly (Rothaermel and Hill 2005; Zhu et al. 2019). Meanwhile, production capacity expansion for new products usually requires significant investments in new plants and equipment. Therefore, relatively high firm-level profit may rely on successfully introducing a stream of new products (Artz et al. 2010). Firms need to make iterative improvements to deal with defects and deficiencies of new products, needing further investment on complementary assets (Rothaermel 2001). Considering the huge investment in marketing and complementary resources necessary to overcome the liability of newness and obtain firm-specific advantages (Lee et al. 2021), the increasing yield of new products might lead to higher losses than profits regarding the firm’s financial report before they could “jump out of the valley of innovation” (Barr et al. 2009). Hence, a negative relationship may exist between innovation output and financial performance during this stage.

With the continuous improvement and establishment of the product selling network and supply chain, these complementary assets would generate dynamic capabilities to creatively appropriate economic value from their new products (Lee et al. 2021). This helps improve the efficiency of commercializing new products and obtains marginal benefits from new product sales. With the increasing acceptance of new products, the sales and market share increase and accelerate due to the economics of scale (Guo and Zheng 2019). Therefore, the up-front innovation costs can be continuously decreased. Once the average up-front cost is lower than the marginal benefit of new product sales, the production and sales increase of new products will bring the expected growth of revenue and profit and, therefore, better financial performance. Furthermore, it means a positive follow-up relationship exists between innovation output and financial performance when innovation output passes a certain point. Therefore, the relationship between innovation output and financial performance would not be linear but a curvilinear one. Hence, we propose the following hypothesis:

#### Hypothesis 1

There is a U-shaped relationship between a firm’s innovation output and financial performance, such that financial performance declines at a low innovation output level and subsequently rises as the level of innovation output further increases.

### Moderating effect of IFC

It is considered that financial constraints play an essential role in a firm’s performance. For instance, some scholars have discovered the negative impacts of a firm’s financial constraints on aggregate productivity and total factor productivity (Gorodnichenko and Schnitzer 2013). This research focuses on firm-level financial constraints by differentiating the IFC from MFC. IFC originates from the firm’s specific characteristics, such as size, age, and leverage, and the intensity and degree of IFC may vary across firms (Hottenrott and Peters 2012). Usually, small and young firms often endure severe financial constraints due to fewer advantages in dealing with information asymmetry and agency costs. Moreover, small and young firms have less bargaining power in the collaboration networks or supply chain system, leading to a more volatile growth pattern, bringing significant challenges in accessing financial support (Cleary 2006; Kadapakkam et al. 1998).

In addition to market performance and financial performance, literature has also highlighted the critical role of IFC on organizational risk-taking behaviors, such as building new channels, constructing a new selling network, and conducting marketing projects (Teece 1986, 2018). Some scholars focus on the costs of funds resulting from IFC, which influence all the innovation dimensions, followed by the economic performance (Hall et al. 2015). In some cases, innovating firms may suspend their innovation commercialization projects, such as marketing and network building, due to the lack of external funds (Hall et al. 2015). Such projects would be profitable at the internal rate of return but are not rewarding given the risk-premium on the costs of external capital.

Thus, IFC may restrain firms’ capabilities to improve their complementary resources to overcome the liability of newness and commercialize their innovation outputs. The innovating firms find it more difficult to enhance their financial performance through innovation output due to the higher costs resulting from the severe IFC. Thus, IFC negatively impacts a firm’s profit from its innovation output. Therefore, we propose the following hypothesis:

#### Hypothesis 2

The U-shaped relationship between innovation output and financial performance is attenuated by the IFC.

### Moderated effect of MFC

The financial market is a very complex system (Zha et al. 2020) and plays an essential role in promoting technological innovation and economic development (Schumpeter 1911). However, due to market imperfections and turbulence, financial constraints can be external. Current literature highlights the crucial role of external funding channels that result from financial developments in innovation (Benfratello et al. 2008; Brown et al. 2012; Cornaggia et al. 2012; Mayer and Sussman 2005). However, such channels are embedded in the environment and networks where innovative firms run their business. Such MFC results from the underdevelopment and imperfection of the financial market. When the environment becomes unfavorable under severe MFC, decision-makers become less prone to engaging in innovation inputs and activities that commercialize their innovation outputs due to high costs and low credit availability (Guiso et al. 2006; Lopezmartin 2017). The lack of credit availability can constrain resource allocation and reduce firm-level investment, especially in transition economies (Paravisini 2008). In such underdeveloped financial environments, investors may be less motivated to support innovative firms due to the difficulty in evaluating the potential economic value and financial returns of the firm’s new products or technologies (Wurgler 2001).

In developed financial markets, financial intermediaries and professional service providers can facilitate investments (both tangible and intangible), motivating firms to pursue innovation and leveraging these intermediaries to diffuse their innovation outputs (King and Levine 1993). Therefore, the development of financial markets can alleviate MFC, thus motivating entrepreneurship (Black and Strahan 2002), increasing credit supply (Cetorelli and Strahan 2006; Guiso et al. 2006; Rice and Strahan 2010), promoting creative destruction (Kerr and Nanda 2009) and increasing innovation output (Benfratello et al. 2008; Cornaggia et al. 2012). Therefore, underdevelopment in capital allocation due to the MFC is strongly associated with a lower degree of both innovation output and firm’s profiting from innovation (Beck et al. 2004; Chemmanur et al. 2014). Thus, MFC may limit the firm’s profitability and negatively impact firms benefiting from innovation output. Therefore, we propose the following hypothesis:

#### Hypothesis 3

The U-shaped relationship between innovation output and financial performance is attenuated by the MFC.

### Joint moderating effect of IFC and MFC

The MFC reductions might indirectly affect the profiting process of innovation by releasing the level of IFC. Thus, the joint moderating effect of IFC and MFC may exist. The improvements in financial market functioning reduce firms’ perceived financial constraints (Love 2003). With the development of financial markets, the financial intermediaries’ capabilities to collect and analyze information will also increase, leading to better assessment, selection, and monitoring of investment projects. Such improvements will further facilitate the migration of funds to move towards the highest social and economic returns (Boyd and Prescott 1985; Greenwood and Jovanovic 1990). The alleviation of MFC results in lower cost and easier access to external funds for firms under severe IFC, with more salient influences on the small and young firms looking for resources to commercialize their innovation, including new technologies and its corresponding yields. Moreover, capital allocations will be more efficient with such improvements, followed by better investment opportunities and actual growth (Cleary et al. 2007). Hence, the alleviation of MFC will benefit firms that suffer more from IFC. In other words, a higher level of MFC will suppress firms that suffer more from IFC to restrain the firm’s profiting from innovation indirectly.

Furthermore, firms face challenges in improving the pace of their productivity and innovation commercialization without a supportive financial system investing in complementary assets (Fazzari et al. 1988). When MFC is severe, innovative firms under high IFC pay an even higher premium for externally raised funds over internally generated funds. Therefore, when short of funds, firms may be less likely to take risks investing in building sales networks or withdrawing from marketing programs critical for capturing values from their innovation output. Such attitudes may distort the efficiency of firm-level resource allocation, followed by a decrease in the firms’ ability to profit from the innovation and overall productivity (Cleary et al. 2007), creating a collective negative impact on firms’ final economic benefit from their innovation. Hence, even though innovations may have the potential to bring better financial performance, the high costs resulting from the severe MFC will make it even harder for firms under higher IFC to dodge innovation traps and further improve their financial performance. Therefore, we propose the following hypothesis:

#### Hypothesis 4

The IFC and MFC have a negative joint moderating effect on the relationship between innovation output and financial performance.

**Fig. 1:**
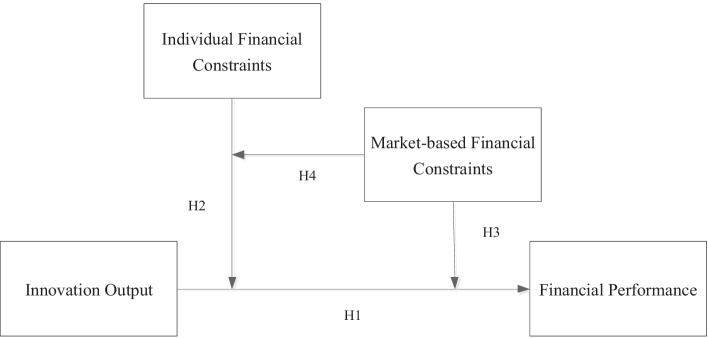
Conceptual framework

The conceptual framework of this research is shown as Fig. 1.

## Methodology

### Data and sample

We employ a multi-source dataset to test our hypothesis empirically. The primary data source of this study is the Annual Industrial Survey Database (1999–2009) of the Chinese National Bureau of Statistics (CNBS). This database contains the most comprehensive information about domestic and foreign firms in China (Tian 2007), and collects firms’ critical financial information such as sales, capital, employment, and demographic information such as ownership and the year the firm was founded (Zhang et al. 2010). It also covers all state-owned and non-state-owned firms (including foreign-invested firms) from 1998, having annual sales of 5 million RMB (about US$720,000 according to the official exchange rate of 2018) or above.

Our sample consists of 142,972 domestic firm-year observations, covering 79,570 domestic firms in manufacturing industries (unevenly distributed across years) that have declared new product sales from 1999–2009. Moreover, the sample data covers 31 Chinese mainland provincial areas (provinces and municipalities directly under the Chinese Central Government, including Beijing, Shanghai, Tianjin, and Chongqing), with 29,065 observations of state-owned firms and 51,679 observations of private firms. From the regional distribution perspective, there are 89,541 observations from the eastern region, 26,350 from the central region, 16,120 from the western region, and 10,964 from the northeast region. Such a complete dataset provides an ideal research setting to examine our theoretical postulations on the relationship between innovation output and financial performance.

### Operationalization of key variables

#### Financial performance

The widely accepted financial performance indicators are income indicators such as primary business revenue, profits, and others. However, considering that the improvement of innovation output can increase sales revenue and improve the firm’s profitability, it may be more accurate to measure financial performance using the profit index (Li and Vermeulen 2021; Teece 1986; Yu et al. 2017). Therefore, we chose the firm’s annual main business profit to indicate the firm’s financial performance level.

#### Innovation output

It is widely recognized that R&D does not capture all aspects of innovation that can often occur through other channels. Recent studies have shifted the definition of innovation activities from an input perspective to an output approach by including the outcome of the innovation process in the regressions rather than in its input (Hall et al. 2009). Training, technology adoption, patents, and sales of products new to the firm's market are the proposed indicators to measure innovation output. We take the logarithm of the values of firm’s new products to measure its innovation output.

#### Individual financial constraints (IFC)

Concerning the endogeneity of leverage and cash flow, some scholars advocate a conservative approach using only firm size and age (SA Index) to create a measurement of financial constraints (Hadlock and Pierce 2010). We use the SA index method to measure the level of IFC. The index is calculated as:

$$IFC = - 0.737 \times Size + 0.043 \times Size^{2} - 0.04 \times Age$$

with *Size* representing the firm size and *Age* representing firm age. Following existing literature, this study uses the logarithm of the firm’s total assets to measure the firm size. It uses the difference between the observation and the registration years to measure the firm age. The SA index is always negative, and the larger absolute value indicates a higher level of IFC.

#### Market-based financial constraints (MFC)

We operationalize the MFC with a provincial index adopted from the marketization indices developed by the National Economic Research Institute of China (Fan et al. 2010). “The indices reflect the development status of market trading mechanisms and other institutions in achieving more efficient market functioning” (Gao et al. 2010). The indices have been widely used in economics, management, and finance studies (Gao et al. 2010; Li et al. 2010). The index we adopted to measure the MFC represented the indices of marketization of the financial industry in each province.

### Control variables

We explored the viability of several control variables that could provide alternative explanations for the hypothesized relationships among the constructs in our model (Table 1):

#### Capital intensity

Capital-intensive firms may focus more on R&D activities and innovation than labor-intensive firms (Chang et al. 2013). We use the proportion of fixed assets to total assets to measure the firm’s capital intensity.

#### Market share

The impact of innovation on market value is more significant for firms with a higher market share (Blundell et al. 2010). We measure market share through the firm’s sales to total industry sales ratio.

#### Subsidy income

Government subsidies might significantly impact a firm’s innovation behaviors (González and Pazó 2008). Therefore, we choose the logarithm of firm subsidy income to measure this control variable.

#### Agency cost

Agency costs in emerging markets can significantly influence a firm’s innovation activities (Chen et al. 2016). Agency cost in this research is measured by the ratio of management expenses to annual sales (Leland 2010).

#### Degree of internationalization

The relationship between internationalization and innovation activities has received wide attention from scholars in recent years. Internationalization can enhance firms’ innovation capability and thus improve their innovation performance (Kafouros et al. 2008). We use a dummy variable to measure a firm’s degree of internationalization with a value of 1 for the firms which export, or 0 for those that do not.

Alongside China’s dramatic economic growth, it is likely that firms’ innovation capabilities have also changed extensively over the past decades (Zhang et al. 2010). To capture this possible effect, we included *year* dummies with 1999 as the base. In addition, we also controlled different *industries*, different *regions*, the *scale,* and *ownership*, to reflect differences in innovation activities between firms.

**Table 1:** Variables description summary

Type	Name	Symbol	Explanation and measurement
Dependent variable	*Financial performance*	Finan	The firm's annual main business profit
Independent variable	*Innovation output*	Innov	The logarithm of the firm’s new products value
Moderation variable	*Individual financial constraints*	IFC	The SA index method(Hadlock and Pierce 2010), the larger absolute value indicates a higher level of IFC
	*Market-based financial constraints*	MFC	Marketization indices of the financial industry in each province developed by the National Economic Research Institute (NERI) of China (Fan et al. 2010)
Control Variables	*Capital intensity*	CI	The proportion of fixed assets to total assets
	*Market share*	Share	The proportion of the firm’s sales to total industry sales
	*Subsidy income*	Subsidy	The logarithm of firm’s government subsidy income
	*Agency cost*	AC	The ratio of management expenses to annual sales (Leland 2010)
	*Internationalization*	Export	Firm’s degree of internationalization with a value of 1 for the firms which export or 0 if not

### Empirical model

The following model is constructed to test the U-shaped impact of innovation output on financial performance:

1 $$perf_{it} =\upbeta _{0} +\upbeta _{1} controls_{{_{it} }} +\upbeta _{2} INNOV_{{_{it} }} +\upbeta _{3} INNOV_{{_{it} }}^{2} + u_{it}$$

Among them, the subscripts *i* and *t* represent the firm and year respectively, *Perf* represents the level of financial performance; *INNOV* represents innovation output; *INNOV*_*it*_^*2*^ represents the square term of *INNOV*; *Controls*_*it*_ represents factors such as the year, region, industry, and scale that affect financial performance; *u*_*it*_ represents random Errors.

We further add the interaction term *INNOV*_*it*_ × *IFC*, *INNOV*_*it*_^*2*^ × *IFC, INNOV*_*it*_ × *MFC,* and *INNOV*_*it*_^*2*^ × *MFC* to investigate the moderating effect of IFC and MFC on the U-shaped relationship between innovation output and financial performance.

2 $$\begin{aligned} perf_{it} & =\upbeta _{0} +\upbeta _{1} controls_{it} +\upbeta _{2} INNOV_{it} +\upbeta _{3} INNOV_{it}^{2}+\upbeta _{4} IFC +\upbeta _{5} INNOV_{it} \times IFC \\ & \quad +\upbeta _{6} INNOV_{{_{it} }}^{2} \times IFC +\upbeta _{7} MFC +\upbeta _{8} INNOV_{{_{it} }} \times MFC +\upbeta _{9} INNOV_{{_{it} }}^{2} \times MFC + u_{it} \\ \end{aligned}$$

To test the combined moderating effect of IFC and MFC, we then increase the interaction term *INNOV*_*it*_ × *IFC* × *MFC* and *INNOV*_*it*_^*2*^ × *IFC* × *MFC* in the Model:

3 $$\begin{aligned} perf_{it} & =\upbeta _{0} +\upbeta _{1} controls_{{_{it} }} +\upbeta _{2} INNOV_{{_{it} }} +\upbeta _{3} INNOV_{{_{it} }}^{2} +\upbeta _{4} IFC +\upbeta _{5} INNOV_{{_{it} }} \times IFC \\ & \quad +\upbeta _{6} INNOV_{{_{it} }}^{2} \times IFC +\upbeta _{7} MFC +\upbeta _{8} INNOV_{{_{it} }} \times MFC +\upbeta _{9} INNOV_{{_{it} }}^{2} \times MFC \\ & \quad +\upbeta _{10} IFC \times MFC +\upbeta _{11} INNOV_{{_{it} }} \times IFC \times MFC +\upbeta _{12} INNOV_{{_{it} }}^{2} \times IFC \times MFC + u_{it} \\ \end{aligned}$$

We first use the panel ordinary linear square regression (Panel OLS) method to test our theoretical postulations using the above empirical models. As some other factors cannot always be observed and innovation activities are highly heterogeneous among industries, fixed-effects models can reduce the impact of heterogeneity and missing variables. We also consider the year-fixed effect of controlling the general external influence of economic dynamics or shocks such as the financial crisis of 2008. We also use the fixed-effect model as a robustness check.

The software we use for regressions and hypothesis testing is STATA 16.0.

## Results

### Descriptive statistics

Table 2 shows descriptive statistics and intercorrelations among the study variables (except year, region, ownership, and industry dummies). Financial performance is positively related to innovation output (*r* = 0.512, *p* < 0.01), IFC (*r* = 0.387, *p* < 0.01), and MFC (*r* = 0.081, *p* < 0.01). The control variables, market share, subsidy, and degree of internationalization, have positive relationships with financial performance and innovation output. However, capital intensity and agency costs have negative relationships with financial performance and innovation output.

**Table 2:** Descriptive statistics and correlations among main variables

	Variables	M	SD	1	2	3	4	5	6	7	8
1	Finan	0.6937	1.471	1							
2	Innov	9.277	2.127	0.512****	1						
3	IFC	− 3.361	0.604	0.387***	0.360***	1					
4	MFC	8.687	2.637	0.081***	0.115***	0.203***	1				
5	Share	0.002	0.012	0.210***	0.160***	0.130 ***	− 0.050***	1			
6	CI	0.333	0.195	− 0.053***	− 0.080***	− 0.025***	− 0.116***	0.040**	1		
7	AC	0.106	1.179	− 0.023***	− 0.030***	− 0.031***	− 0.033***	− 0.006***	0.001	1	
8	Subsidy	1.200	2.568	0.162***	0.217***	0.098***	− 0.044	0.092***	− 0.025***	0.001	1
9	Export	0.528	0.499	0.136***	0.114***	0.096***	0.124***	0.050***	0.001	− 0.017***	0.099***

n = 142,975. Finan = Financial performance; Innov = Innovation output; IFC = Individual financial constraints; MFC = Market-based financial constraints; Share = Market Share; CI = Capital Intensity; AC = Agency Costs; Subsidy = Subsidy Income; Export for 1 “Yes” and 0 for “No”; *p < 0.1, ** p < 0.05, *** p < 0.01

### Hypotheses tests

Table 3 presents the hierarchical multiple regression results. The results of Model 1 suggest that the market share in the industry is positive and significant (*b* = 0.130 *p* < 0.01), and the degree of internationalization is also positive and significant (*b* = 0.111, *p* < 0.01). Moreover, capital intensity is negative and significant (*b* =  − 0.248, *p* < 0.01), and the first-order effect of the innovation output is positive and significant (*b* = 0.226, *p* < 0.01). Model 2 adds the squared term of innovation output, which is positive and significant (*b* = 0.057, *p* < 0.01), while its first-order becomes negative and significant (*b* = − 0.770, *p* < 0.01). These results show that hypothesis 1 is not rejected and firm innovation output has a U-shaped relationship with financial performance.

**Table 3:** Main hypotheses testing results

Independent variables	Panel OLS				FE	RE
	Model 1	Model 2	Model 3	Model 4	Model 5	Model 6
*Share*	0.130***	0.093***	0.082***	0.082***	0.049***	0.082***
	(6.20)	(5.90)	(5.66)	(5.64)	(9.58)	(28.41)
*CI*	− 0.248***	− 0.202***	− 0.220***	− 0.222***	− 0.258***	− 0.222***
	(− 13.52)	(− 11.76)	(− 12.95)	(− 13.13)	(− 7.86)	(− 13.78)
*Cost*	− 0.005	− 0.008*	− 0.008*	− 0.008*	− 0.005	− 0.008***
	(− 1.36)	(− 1.71)	(− 1.85)	(− 1.81)	(− 1.53)	(− 3.48)
*Subsidy*	0.011***	0.007***	0.006***	0.005***	− 0.004**	0.005***
	(6.28)	(3.79)	(3.34)	(2.97)	(− 2.21)	(4.33)
*DOI*	0.111***	0.079***	0.077***	0.076***	0.024*	0.076***
	(13.77)	(10.55)	(10.47)	(10.35)	(1.80)	(11.31)
*Innov*	0.226***	− 0.770***	− 0.054	− 1.337***	− 1.874***	− 1.337***
	(85.36)	(− 59.03)	(− 0.63)	(− 7.81)	(− 10.36)	(− 12.09)
*Innov*^*2*^		0.057***	0.025***	0.100***	0.121***	0.100***
		(70.34)	(5.19)	(10.33)	(13.92)	(18.01)
*IFC*			− 0.419***	0.962***	1.618***	0.962***
			(− 4.97)	(5.06)	(6.40)	(6.59)
*IFC* × *Innov*			0.102***	− 0.295***	− 0.422***	− 0.295***
			(5.21)	(− 6.39)	(− 8.51)	(− 9.68)
*IFC* × *Innov*^*2*^			− 0.002*	0.021***	0.027***	0.021***
			(− 1.86)	(7.79)	(10.66)	(13.38)
*MFC*			− 0.419***	0.962***	1.618***	0.962***
			(− 4.97)	(5.06)	(6.40)	(6.59)
*MFC* × *Innov*			− 0.024***	0.134***	0.150***	0.134***
			(− 5.62)	(6.59)	(7.22)	(10.06)
*MFC* × *Innov*^*2*^			0.002***	− 0.008***	− 0.009***	− 0.008***
			(6.05)	(− 6.93)	(− 9.36)	(− 11.65)
*IFC* × *MFC*				− 0.172***	− 0.161***	− 0.172***
				(− 6.80)	(− 5.23)	(− 9.15)
*IFC* × *MFC* × *Innov*				0.050***	0.050***	0.050***
				(8.62)	(8.43)	(13.04)
*IFC* × *MFC* × *Innov*^*2*^				− 0.003***	− 0.003***	− 0.003***
				(− 9.03)	(− 10.73)	(− 15.13)
*_cons*	− 2.114***	2.201***	− 0.451	3.979***	6.553***	3.979***
	(− 30.24)	(26.57)	(− 1.20)	(5.45)	(6.03)	(7.27)
*Year FE*	Yes	Yes	Yes	Yes	Yes	Yes
*Industry FE*	Yes	Yes	Yes	Yes	Yes	Yes
*Scale*	Yes	Yes	Yes	Yes	Yes	Yes
*Area FE*	Yes	Yes	Yes	Yes	Yes	Yes
*Ownership*	Yes	Yes	Yes	Yes	Yes	Yes
*N*	142,975	142,975	142,972	142,972	142,972	142,972
*chi*^*2*^	17,914.1	23,979.2	26,704.7	27,431.2	–	82,709.9
*r2_b*	0.350	0.432	0.455	0.456	0.223	0.456
*r2_o*	0.356	0.435	0.455	0.456	0.255	0.456

*p < 0.1; **p < 0.05; ***p < 0.01 Two-tailed tests

Hypothesis 2 proposes that innovation output can bring better financial performance for firms facing low IFC. Model 3 includes the interaction term of innovation output and IFC. As discussed above, the SA index is always negative, and the larger absolute value indicates higher IFC. The coefficient of the first-order interaction term *IFC* × *Innov* (*b* = 0.102, *p* < 0.01) is positive and significant, while the coefficient of second-order interaction term *IFC* × *Innov*^*2*^ (*b* = − 0.002, *p* < 0.1) is negative and significant. These results are consistent with hypothesis 2.

**Fig. 2:**
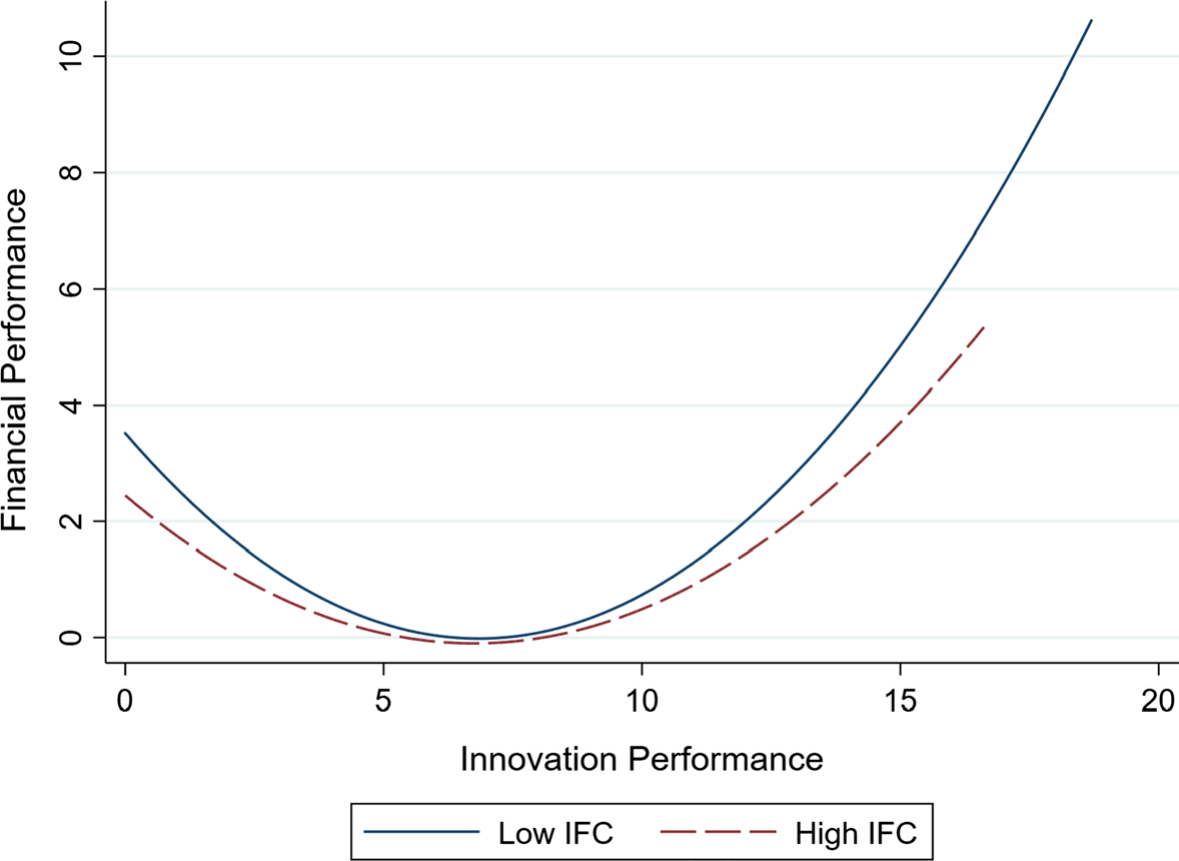
Relationship between innovation output and financial performance under different levels of individual financial constraints

To facilitate interpretation, we plotted the U-shaped relationship curve between innovation output and financial performance under different levels of IFC in Model 3. The observations are divided into two groups based on the mean of IFC to illustrate our results. As shown in Fig. 2, the curve of innovation output and financial performance is higher when facing low IFC than when facing high IFC. To further make the moderating effects clear, we calculate the average marginal effects under different levels of IFC.

As shown in Table 4, when firms face low IFC, the average marginal effect of innovation output on financial performance is 0.333 (*p* < 0.01). When firms face high IFC, the corresponding average marginal effect is 0.186 (*p* < 0.01). These results indicate that hypothesis 2 is supported.

**Table 4:** Average marginal effects of innovation output on financial performance under different levels of financial constraints

Group	n	IFC	MFC	AME	Std Err	z	*p* > z	95% Conf. Interval	
1	85,882	Low	–	0.333	0.004	82.440	0.000	0.325	0.341
2	57,093	High	–	0.186	0.004	47.920	0.000	0.179	0.194
3	72,698	–	Low	0.307	0.005	66.850	0.000	0.298	0.316
4	70,277	–	High	0.258	0.004	66.900	0.000	0.250	0.265
5	47,677	Low	Low	0.348	0.006	58.280	0.000	0.336	0.360
6	38,205	Low	High	0.302	0.006	54.440	0.000	0.291	0.313
7	25,021	High	Low	0.193	0.006	32.800	0.000	0.181	0.204
8	32,072	High	High	0.167	0.005	32.550	0.000	0.157	0.177

AME = Average Marginal Effect; IFC = Individual financial constraints; MFC = Market-based Financial Constraints

Hypothesis 3 proposes that financial performance is better for firms under low MFC than those under high MFC. Model 3 also includes the interaction term of innovation output and MFC. The coefficient of the first-order interaction term *MFC* × *Innov* (*b* = − 0.024, *p* < 0.01) is negative and significant, while the coefficient of the second-order interaction term *MFC* × *Innov*^*2*^ (*b* = 0.002, *p* < 0.01) is positive and significant. These results are consistent with hypothesis 3.

**Fig. 3:**
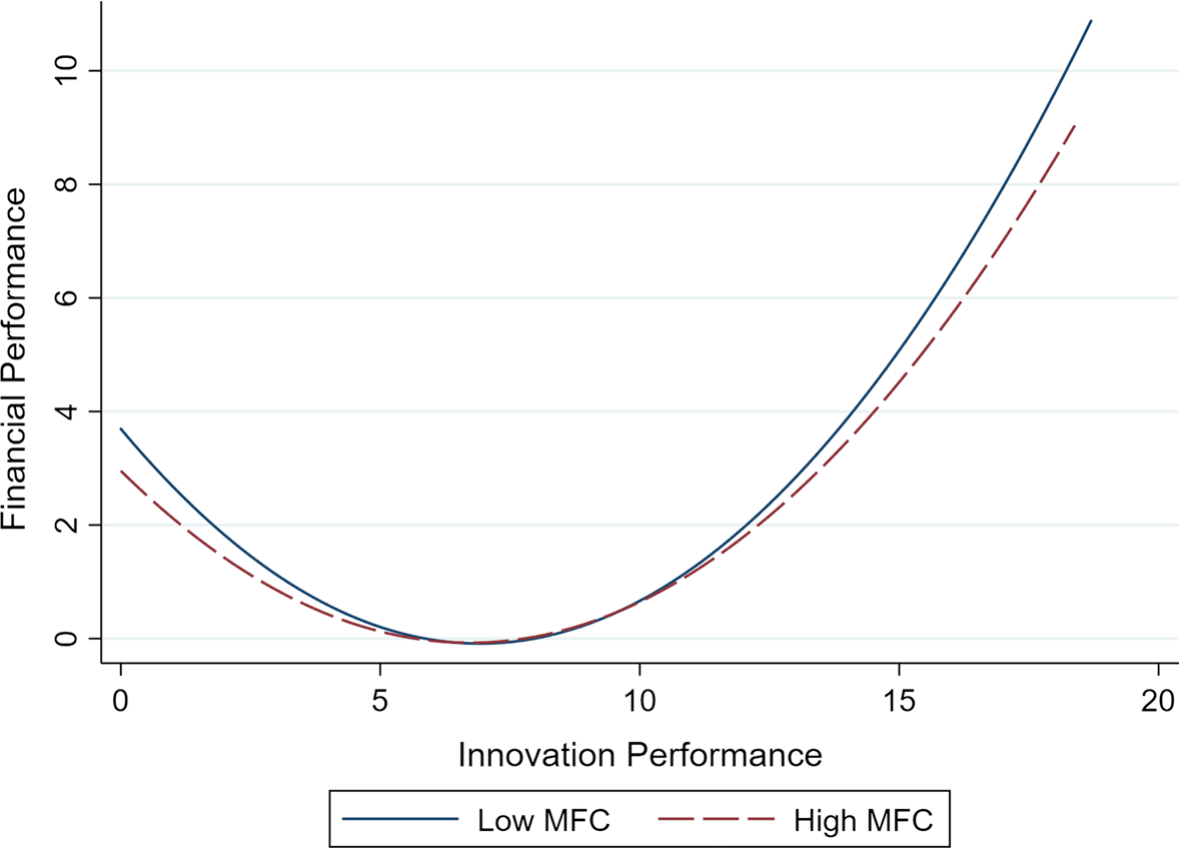
Relationship between innovation output and financial performance under different levels of market-based financial constraints

We plotted the U-shaped relationship curve between innovation output and financial performance under different levels of MFC in Model 3 to aid interpretation. The observations are divided into two groups based on the mean of MFC. As shown in Fig. 3, the curve of innovation output and financial performance is higher when facing low MFC than when facing high MFC. To further clarify the moderating effect, we calculated the average marginal effect of innovation output on financial performance under different levels of MFC. As shown in Table 4, when firms face low MFC, the average marginal effect of innovation output on financial performance is 0.307 (*p* < 0.01). In comparison, when firms face high MFC, the corresponding average marginal effect is 0.258 (*p* < 0.01). Thus hypothesis 3 is supported.

**Fig. 4:**
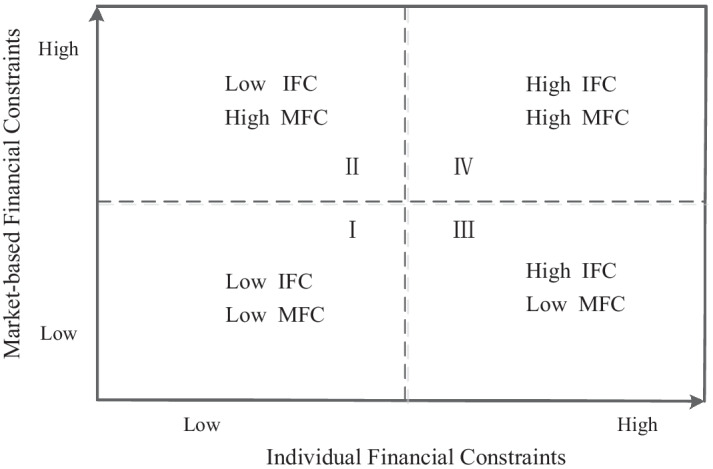
Different types of financial constraints

To better understand the different impacts of financial constraints on the relationship between innovation output and financial performance, we examined the joint moderating effect of IFC and MFC. Model 4 includes the interaction term of innovation output, IFC, and MFC. The coefficient of the interaction term *IFC* × *MFC* × *Innov* (*b* = 0.050, *p* < 0.01) is positive and significant, and the coefficient of the interaction term *IFC* × *MFC* × *Innov*^*2*^ (*b* = − 0.003, *p* < 0.01) is negative and significant. As shown in Fig. 4, the observations in our research are divided into four groups by the mean of IFC and MFC.

**Fig. 5:**
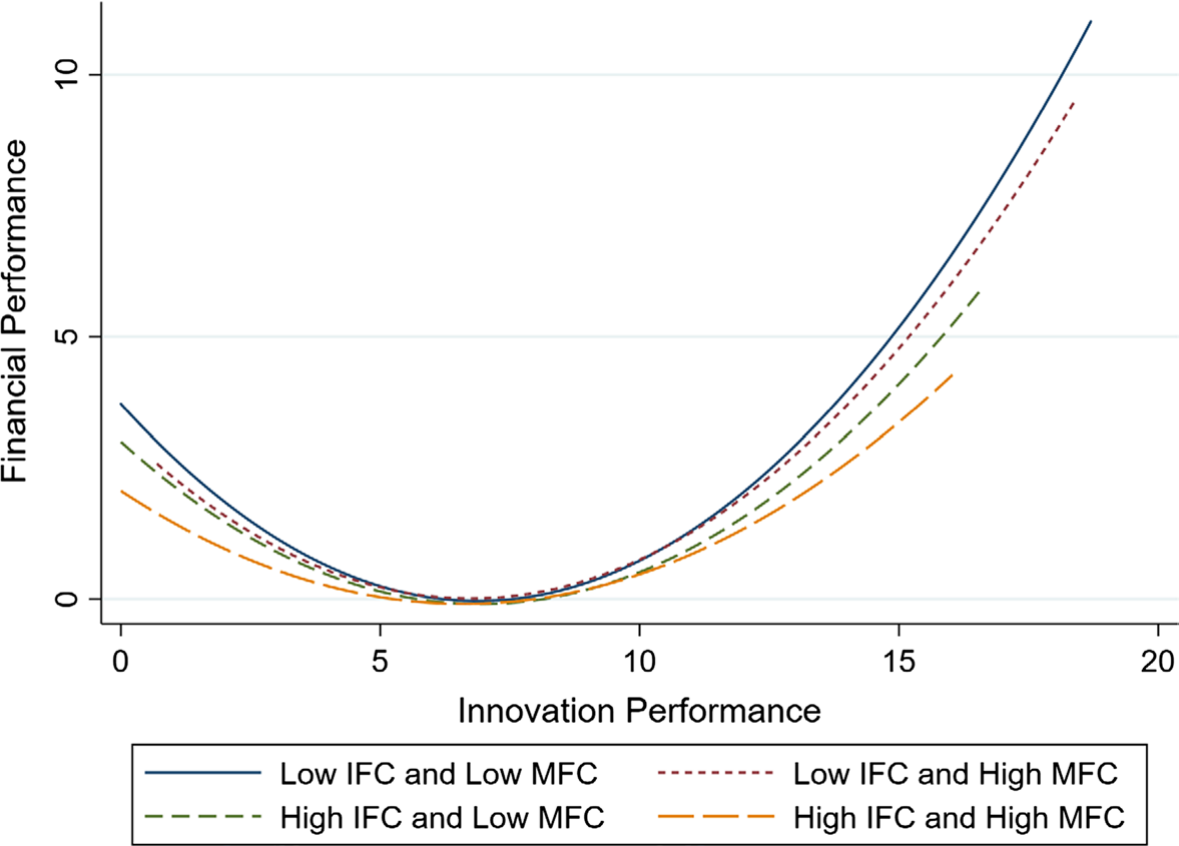
Relationship between innovation output and financial performance under different levels of financial constraints

The observations can be categorized into four distinct types: low IFC and low MFC group, low IFC and high MFC, high IFC and low MFC group, and high IFC and high MFC group. To understand such results, we plotted the U-shaped relationship curve between innovation output and financial performance under different types of financial constraints, as shown in Fig. 5.

Moreover, to illustrate the joint moderating effects, we calculated the corresponding average marginal effects. As shown in Table 4 above, when IFC and MFC are both low, the average marginal effect of innovation output on financial performance is 0.348 (*p* < 0.01). In comparison, when the two financial constraints are both high, the corresponding average marginal effect is 0.167 (*p* < 0.01). However, when IFC is low and MFC is high, the marginal effect is 0.302 (*p* < 0.01); and when IFC is high and MFC is low, the marginal effect is 0.193 (*p* < 0.01). These results are consistent with hypothesis 3.

### Robustness test and additional analysis

This study aims to examine the effects of firms’ innovation output on their financial performance. A possible reverse causality may exist; firms with better financial performance may bring superior innovation output. Therefore, firms’ innovation output may be partially determined by their financial performance. We conducted the following endogeneity check to rule out such reverse causality (Belderbos et al. 2013; Zhang et al. 2010). We regressed the average change of innovation output from year *t*− 1 to year *t* on the average financial performance of all firms in the industry in year *t*− 1*.* The coefficient of financial performance is 0.004 (p < 0.951). We also regressed the financial performance in year *t* on the average financial performance of all firms in the industry in year *t*− 1, and the coefficient of financial performance is 0.106 (p < 0.179). If any one of the predictors had been significant, it would raise endogeneity concerns. However, our results showed that none of these predictors was significant. Thus, we concluded that the likelihood of such reverse causality is very low.

As a robustness check, we further introduced the mean of the annual innovation output by the industry as an instrumental variable (IV) (Clausen 2009; Heutel 2009). The premise of using the instrumental variable method is that there is an endogenous explanatory variable; the Chi-sq(1) in the endogeneity test of endogenous regressors is 430.90 (*p* < 0.01), which can significantly reject innovative output as an exogenous variable. We also used various statistical tests to check the validity of the instrumental variables. The under-identification test showed that the Kleibergen-Paap rk LM statistic was 256.100 (*p* < 0.001). The weak identification test showed that the Cragg-Donald Wald F statistic was 167.782, and the Kleibergen-Paaprk Wald F statistic was 131.649, higher than the 10% maximal IV size (10% maximal IV size: 7.03). As the number of endogenous variables and instrumental variables is equal, the over-identification problem does not exist. Based on the analysis above, the choice of the instrumental variables in this study is reasonable. Comparing the results of the panel OLS and fixed effect estimation, we can conclude that the coefficient and significance level of the key variables in this study are consistent, further verifying the robustness of our results.

Since China implemented a new National Economic Industry Classification in 2003, we chose the Annual Industrial Survey Database data from 2003–2009 for consistent industry classification when re-checking our hypotheses. Therefore, it is reasonable to believe that the instrumental variables are not weak in our research. Considering the heteroscedasticity concerns, we also implemented the Generalized Method of Moments (GMM) method, more efficient in processing heteroscedasticity. As shown in Table 5, the regression results are entirely consistent with our hypotheses.

**Table 5:** Regression analysis results using data from 2003 to 2009

Independent variables	Panel OLS	FE	RE	GMM
	Model 7	Model 8	Model 9	Model 10
*Share*	0.0819***	0.0551***	0.0551***	0.0282
	(4.08)	(7.57)	(7.57)	(1.31)
*CI*	− 0.2041***	− 0.2186***	− 0.2186***	− 0.1122***
	(− 10.99)	(− 5.89)	(− 5.89)	(− 2.68)
*Cost*	− 0.0101*	− 0.0079	− 0.0079	− 0.0246
	(− 1.71)	(− 1.37)	(− 1.37)	(− 1.26)
*Subsidy*	0.0074***	− 0.0037**	− 0.0037**	− 0.0020
	(3.82)	(− 1.97)	(− 1.97)	(− 0.42)
*DOI*	0.0879***	0.0401**	0.0401**	0.0004
	(10.43)	(2.45)	(2.45)	(0.01)
*Innov*	− 0.9306***	− 2.2851***	− 2.2851***	1.6011**
	(− 2.81)	(− 6.72)	(− 6.72)	(2.55)
*Innov*^*2*^	0.0743***	0.1452***	0.1452***	− 0.1576**
	(4.21)	(9.17)	(9.17)	(− 2.52)
*IFC*	− 0.1894**	− 0.5730***	− 0.5730***	2.9434***
	(− 1.96)	(− 5.63)	(− 5.63)	(3.33)
*IFC* × *Innov*	0.0143***	0.0363***	0.0363***	− 0.1887***
	(2.69)	(7.42)	(7.42)	(− 3.29)
*IFC* × *Innov*^*2*^	0.5796	2.0685***	2.0685***	− 10.3073***
	(1.37)	(3.89)	(3.89)	(− 3.34)
*MFC*	0.1016***	0.1847***	0.1847***	− 0.2618*
	(2.88)	(5.20)	(5.20)	(− 1.86)
*MFC* × *Innov*	− 0.0055***	− 0.0112***	− 0.0112***	0.0192**
	(− 2.99)	(− 6.87)	(− 6.87)	(2.39)
*MFC* × *Innov*^*2*^	− 0.2491	− 0.4740**	− 0.4740**	0.8662
	(− 1.52)	(− 2.47)	(− 2.47)	(1.29)
*IFC* × *MFC*	− 0.1382***	− 0.1859***	− 0.1859***	0.7565**
	(− 2.93)	(− 3.29)	(− 3.29)	(2.33)
*IFC* × *MFC* × *Innov*	0.0407***	0.0613***	0.0613***	− 0.1993**
	(3.91)	(5.72)	(5.72)	(− 2.51)
*IFC* × *MFC* × *Innov*^*2*^	− 0.0023***	− 0.0038***	− 0.0038***	0.0125***
	(− 4.09)	(− 7.57)	(− 7.57)	(2.64)
*Year FE*	Yes	Yes	Yes	Yes
*Industry FE*	Yes	Yes	Yes	Yes
*Scale*	Yes	Yes	Yes	Yes
*Area FE*	Yes	Yes	Yes	Yes
*Ownership*	Yes	Yes	Yes	Yes
*N*	142,975	142,975	142,975	142,975
*chi*^*2*^	17,914.1	23,979.2	26,284.3	26,322.0
*r2_b*	0.350	0.432	0.453	0.453
*r2_o*	0.356	0.435	0.453	0.453

*p < 0.1; **p < 0.05; ***p < 0.01; Two-tailed tests

From the ownership perspective, technology level, management efficiency, and policy dividend may significantly differ among firms with different types of ownership. Different scales mean different organizational characteristics and resources, and big firms possessing abundant resources can effectively buffer the impacts of external environmental changes. We check these questions and list the corresponding subgroup regression results. As shown in Table 6, the regression results are basically consistent with the hypotheses.

**Table 6:** Subgroup regression results of firms under different ownership and different scale

Independent variables	Scale		Ownership		Area	
	large	SMEs	SOEs	Non-SOEs	East	Non-East
	Model 11	Model 12	Model 13	Model 14	Model 15	Model 16
*Share*	0.085***	0.028***	0.032***	0.045***	0.064***	0.025***
	(6.02)	(5.41)	(2.65)	(8.20)	(9.88)	(2.94)
*CI*	− 0.899***	− 0.170***	− 0.577***	− 0.171***	− 0.335***	− 0.169***
	(− 5.56)	(− 6.23)	(− 5.99)	(− 5.15)	(− 7.88)	(− 3.23)
*Cost*	− 0.345***	− 0.003	− 0.003	− 0.008	− 0.004	− 0.010
	(− 4.60)	(− 1.19)	(− 0.77)	(− 1.63)	(− 1.21)	(− 1.32)
*Subsidy*	− 0.018***	0.001	− 0.020***	0.004**	− 0.001	− 0.009***
	(− 3.87)	(0.77)	(− 5.97)	(2.47)	(− 0.35)	(− 3.35)
*DOI*	0.068	0.005	0.043	0.020	0.007	0.073***
	(1.23)	(0.46)	(1.34)	(1.36)	(0.41)	(3.15)
*Innov*	− 1.299**	− 1.201***	− 1.483***	− 2.262***	− 2.359***	− 1.825***
	(− 2.03)	(− 5.66)	(− 4.39)	(− 9.19)	(− 8.35)	(− 7.01)
*Innov*^*2*^	0.081***	0.092***	0.094***	0.151***	0.142***	0.117***
	(2.93)	(7.82)	(6.03)	(12.46)	(10.67)	(9.38)
*IFC*	0.842	0.911***	1.359***	1.911***	2.340***	1.576***
	(0.79)	(3.54)	(2.92)	(5.55)	(5.76)	(4.39)
*IFC* × *Innov*	− 0.165	− 0.308***	− 0.297***	− 0.567***	− 0.543***	− 0.418***
	(− 0.87)	(− 5.35)	(− 3.37)	(− 8.19)	(− 6.92)	(− 5.95)
*IFC* × *Innov*^*2*^	0.009	0.023***	0.018***	0.037***	0.032***	0.026***
	(1.03)	(7.08)	(4.21)	(10.52)	(8.26)	(7.53)
*MFC*	− 0.494	0.415***	− 0.381	− 0.437***	− 0.721***	− 0.576***
	(− 1.06)	(3.63)	(− 1.63)	(− 3.09)	(− 4.49)	(− 2.99)
*MFC* × *Innov*	0.154**	− 0.054**	0.113***	0.176***	0.192***	0.167***
	(1.97)	(− 2.21)	(2.72)	(6.43)	(6.41)	(4.73)
*MFC* × *Innov*^*2*^	− 0.009***	0.002*	− 0.007***	− 0.011***	− 0.011***	− 0.010***
	(− 2.69)	(1.82)	(− 3.74)	(− 8.40)	(− 7.71)	(− 6.17)
*IFC* × *MFC*	− 0.211	0.064**	− 0.138**	− 0.170***	− 0.225***	− 0.185***
	(− 1.51)	(2.02)	(− 2.30)	(− 4.25)	(− 5.06)	(− 3.55)
*IFC* × *MFC* × *Innov*	0.052**	− 0.003	0.038***	0.059***	0.059***	0.056***
	(2.15)	(− 0.46)	(3.42)	(7.54)	(6.96)	(5.67)
*IFC* × *MFC* × *Innov*^*2*^	− 0.003***	− 0.000	− 0.002***	− 0.004***	− 0.003***	− 0.003***
	(− 2.68)	(− 0.37)	(− 4.57)	(− 9.62)	(− 8.29)	(− 7.47)
*_cons*	5.000	3.101***	5.631***	6.923***	10.013***	6.161***
	(1.30)	(2.85)	(2.94)	(5.01)	(6.46)	(3.80)
*Year FE*	Yes	Yes	Yes	Yes	Yes	Yes
*Industry FE*	Yes	Yes	Yes	Yes	Yes	Yes
*Scale*	No	No	Yes	Yes	Yes	Yes
*Area FE*	Yes	Yes	Yes	Yes	No	No
*Ownership*	Yes	Yes	No	No	Yes	Yes
N	19,928	123,044	29,064	113,908	89,541	53,431
F	19.996	126.911	26.922	113.580	90.653	63.612
r2_b	0.035	0.175	0.089	0.272	0.299	0.232
r2_o	0.039	0.190	0.100	0.306	0.326	0.264

*t* statistics in parentheses

*p < 0.1; **p < 0.05; ***p < 0.01

In summary, results from the empirical test show that financial performance could not be linearly predicted by innovation output, and it differs among different firms due to the discrepancy between IFC and MFC. Specifically, empirical results indicate that both IFC, measured by the SA index (Hadlock and Pierce 2010), and MFC, measured by the indices of financial industry marketization in each province (Fan et al. 2010), play an important moderating role. Moreover, there are joint moderating effects of the two dimensions of financial constraints on firms’ profiting from innovation. Based on our results, we further classified firms based on these two dimensions into four distinct groups: low IFC and low MFC, low IFC and high MFC, high IFC and low MFC, and high IFC and high MFC. The average marginal effects of innovation output on financial performance significantly differed between the groups, further confirming the main results and our theoretical predictions.

## Discussions

### Contributions

By empirically documenting the curvilinear impact of innovation output on firms’ financial performance and the heterogeneous effect of the individual- and market-based financial constraints, this study contributes to the emerging literature on connecting finance with innovation and firms’ sustainable competitive advantages. Briefly, much discussion on the topic of how financial constraints affect R&D investment exists. However, we extend the studies of how financial constraints affect innovation input such as R&D investment (Cincera and Ravet 2010; Czarnitzki and Hottenrott 2011; Howell 2016; Li 2011) through a holistic innovation output-financial performance logic to open the “black box” between innovation output and firm performance disentangling the role of financial constraints. We further investigate the financial constraints followed by a fine-grained empirical test of the role of IFC and MFC. This allows us to closely examine how different financial constraints influence the relationship between firms’ innovation output and financial performance, individually and jointly. This generates novel insights into the role of financial constraints for firms to adopt the innovation-driven strategy and for policymakers to sustain an innovation-driven economy.

Secondly, we develop the PFI framework (Teece 1986, 2018) and address the crucial role of financial constraints in the relationship between innovation output and financial performance. The findings provide comprehensive and nuanced empirical evidence of a “more innovation, more money” story. Our primary contribution lies in highlighting the mechanism and conditions associated with innovation output and financial performance. Previous research has shown that innovation output has a positive linear relationship with financial performance (Artz et al. 2010). This study finds that this general positive relationship still stands. However, the presumed linear relationship may only partially apply. Due to liability of newness (Cafferata et al. 2009; Gimenez-Fernandez et al. 2020; Yang and Aldrich 2017), the continued investments in marketing networks and other complementary resources along with initial sales of new products may bring even more losses until the firms reach a superior position and achieve the economics of scale in the market place (Cafferata et al. 2009; Gimenez-Fernandez et al. 2020). We believe there should be a curvilinear relationship between firms’ innovation output and financial performance from the mixed empirical results of established studies and theoretical arguments. Our study further empirically tests this proposition and finds strong empirical evidence that such a nonlinear U-shaped relationship could be a serious “innovation trap” that restrains firms’ motivation and ability to benefit from the innovation. As financial performance declines at a low level of innovation output and increases when passing a certain point, both the arguments of “more innovation, more money” and “more innovation, less money” are partially valid. Therefore, this study provides a holistic map for scholars to continue the research on firms’ innovation development.

Furthermore, focusing on the difference between IFC and MFC methodologically contributes to the deep understanding of financial constraints. We have two main aims when measuring financial constraints: first, to derive a time-varying index that allows firms to be more or less constrained in different periods; second, to account for (possible) degrees of financial constraint from both internal and external factors. We claim that the main weaknesses of earlier approaches lie in choosing a single variable while neglecting the external financial environment. Our analysis framework defines financial constraints originating from firm-specific characteristics as “individual financial constraints” and those originating from the external financial environment as “market-based financial constraints.” Little of the work to date has comprehensive measurements of financial constraints, and there is a lack of clear understanding about the mechanisms of how financial constraints affect innovation activities. To some extent, by deconstructing the concept of financial constraints and empirically testing their individual and joint moderating effects, our study provides a new perspective and empirical solution to understand the mixed knowledge from previous studies (Almeida et al. 2013; Brown et al. 2012; Hottenrott and Peters 2012; Mina et al. 2013; Musso and Schiavo 2008).

Practically, this study also generates important insights for policymakers and firm managers to make the best of innovation and continue constructing a mature and pro-innovation financial market for long-term sustainable development. First, our research provides a financing innovation perspective for policymakers to understand firms’ fast growth over the last three decades in China (Allen et al. 2005; Guariglia et al. 2011). As a typical example of emerging markets, China is characterized by a poorly developed financial system. Traditionally, the financial market in China has been characterized by government intervention due to the path-dependence of the centrally planned economy. Thus, the credit allocation was biased towards SOEs and large firms (Gordon and Li 2003). Therefore, financial exclusion becomes the critical constraint for the innovation and development of non-SOEs, especially the small and medium enterprises (SMEs), which are important players in economic growth (Kou et al. 2021b). Our study shows that a reduction in the levels of both IFC and MFC could lower the barriers of accessing the capital necessary for firms to profit from innovation output, therefore increasing the economic returns from innovation. This provides empirical evidence for the qualitative arguments that China is fast catching up in economics and innovation. This is due to the improvement of the national innovation system, including the introduction and continuous improvement of the financial market (Chen et al. 2021).

As widely acknowledged, innovation is the key to long-term sustainable endogenous growth (Arrow 1972; Franko 2010; Romer 1986, 1989); the release of MFC resulting from the institutional change denotes the more inclusive financial market and more efficient credit allocation, inspiring more firms to engage in innovation activities and make it possible for the non-SOEs and SMEs to benefit from innovation. In this case, accompanied by China’s ongoing financial reforms, the SMEs and non-SOEs may face less discrimination and have more opportunities to obtain the financial support of which they have been deprived. Therefore, many non-SOEs and SMEs can overcome the so-called “innovation trap” and benefit more from innovation. Thus, it indicates that financial market improvement in emerging economies like China is one of the, if not the most, important factors for sustained innovation-driven development.

The world has been battling the COVID-19 pandemic since the beginning of 2020, creating financial and psychological distress on sectors and economies (Kou et al. 2021a). Therefore, developing the pro-innovation financial market will light the future of China’s innovation-driven development and other emerging economies. As China is an emerging economy (Yin et al. 2019), our study in the Chinese context generates insights for policymakers in other emerging economies to release both firms’ internal and external financial constraints to accelerate the economic transition towards a high-quality and innovation-driven economy.

### Limitations and future research

Our study also has several limitations, each of which opens an avenue for future research. First, we utilize a convenient method, the provincial marketization level of the financial industry (Fan and Wang 2019), to measure MFC. It is necessary to explore a more effective methodology to measure MFC to develop this theoretical construct. Second, the firm’s profiting from innovation and generating economic and financial returns from innovation outputs are embedded in their innovation life-cycle. Although this study empirically tested the innovation outputs’ curvilinear impact on a firm’s financial performance at the general level, future studies need to investigate how the liability of newness and economies of scale evolve during the process of commercialization of new products and technologies (Gimenez-Fernandez et al. 2020; Rothaermel and Hill 2005) to reveal the dynamics of the PFI framework. Additionally, the top management team (TMT) has been an important factor influencing firms’ strategic decisions (Yin et al. 2019). Thus future research may look at the impact of TMT characteristics, such as gender distribution and overconfidence, on firms’ proactive strategies to deal with financial constraints. Additionally, we took a general approach. Although we did not look into specific industries, future research may look at the impact of innovation output on financial performance moderated by IFC and MFC in specific industries such as the high technology industry and the emerging digital-driven industries, which are forerunners in the frontiers of innovation and profiting from the innovation (Teece 2018).

## Data Availability

The datasets used and analyzed during the current study are available from the corresponding author on reasonable request.
